# Selective Activity of Chrysin-6-*C*-Fucopyranoside from *Cyclanthera pedata* Toward Peroxisome Proliferator-Activated Receptor Gamma

**DOI:** 10.3390/molecules30071626

**Published:** 2025-04-05

**Authors:** Marco Zuccolo, Angela Bassoli, Gigliola Borgonovo, Luca Giupponi, Annamaria Giorgi, Aniello Schiano Moriello, Fabio Arturo Iannotti

**Affiliations:** 1 Centre of Applied Studies for the Sustainable Management and Protection of Mountain Areas-CRC Ge.S.Di.Mont., Università degli Studi di Milano, 25048 Edolo, Italy; marco.zuccolo@unimi.it (M.Z.); luca.giupponi@unimi.it (L.G.); anna.giorgi@unimi.it (A.G.); 2 Department of Agricultural and Environmental Sciences-Production, Landscape and Agroenergy-DiSAA, Università degli Studi di Milano, 20133 Milan, Italy; 3 Department of Food, Environmental and Nutritional Sciences (DeFENS), Università degli Studi di Milano, 20133 Milan, Italy; angela.bassoli@unimi.it; 4 Epitech Group SpA, Saccolongo, 35100 Padova, Italy; aniello.schianomoriello@icb.cnr.it; 5 Institute of Biomolecular Chemistry, National Research Council (ICB-CNR), Via Campi Flegrei 34, 80078 Pozzuoli, Italy

**Keywords:** flavone glycosides, peroxisome proliferator-activated receptors (PPARs), transient receptor potential (TRP) ion channels, *Cyclanthera pedata*

## Abstract

Caigua (*Cyclanthera pedata* (L.) Schrad.) is a traditional herbal remedy traditionally used in Latin America for its health benefits and to treat metabolic disorders, including diabetes. Despite interest in its herbal use, the phytochemical properties of caigua’s secondary metabolites are poorly known. This study aimed to isolate the main flavone glycosides from the leaves of caigua landrace cultivated in the Camonica Valley (Italy) using flash chromatography and evaluate their potential activity toward peroxisome proliferator-activated receptors (PPARs) and transient receptor potential (TRP) ion channels through luciferase and intracellular calcium assays. We found that the caigua species-specific flavone glycoside, chrysin-6-*C*-fucopyranoside, showed potent and selective activity toward PPARγ, with no effects on other PPAR subtypes or TRP channels. These findings indicate that the caigua plant could offer a safer alternative to conventional PPARγ agonists, whose use as antidiabetic drugs is limited by severe side effects that currently restrict the clinical use of conventional PPAR agonists.

## 1. Introduction

Peroxisome proliferator-activated receptors (PPARs) are a subfamily of nuclear receptors that play a central role in regulating various physiological processes, especially those related to metabolism, inflammation, and cell differentiation, functioning as transcription factors of a large number of specific target genes. Given their involvement in lipid and glucose metabolism, PPARs have emerged as promising therapeutic targets for treating diseases such as diabetes, obesity, and cardiovascular disorders [1,2]. The PPAR family consists of three distinct members: PPARα, PPARβ/δ, and PPARγ. Although they share overlapping expression distribution, each has unique functions and ligand affinities [3]. The PPARγ subtype is mainly present in adipocytes, macrophages, monocytes, and intestinal cells. In preadipocytes, activating PPARγ is vital for initiating the differentiation process, which promotes fat storage and supports normal glucose balance. Thus, by ensuring that excess fat is stored in mature adipocytes, PPARγ activation helps to prevent its accumulation in other tissues, which could otherwise harm insulin sensitivity. Additionally, PPARγ directly influences glucose transport by regulating glucose transporter 4 (GLUT4), the key protein for insulin-stimulated glucose uptake primarily in muscle and adipose tissues. This process is crucial for maintaining healthy blood sugar levels and reducing the risk of hyperglycemia [4,5]. Therefore, given its ability to enhance insulin sensitivity and improve glucose uptake, PPARγ is a major therapeutic target for treating type 2 diabetes. Agonists of PPARγ, like thiazolidinediones (TZDs), are prescribed to diabetic patients to improve the responsiveness of muscle and adipose tissue to insulin, thus helping to lower blood sugar levels. Despite their effectiveness, however, the use of TZDs in clinical practice is often limited due to significant side effects, including weight gain, fluid retention, and cardiovascular risks, which result from either off-target interactions or prolonged receptor activation [4,6]. For this reason, there is an urgent need to discover novel classes of PPAR agonists due to the increasing global prevalence of metabolic diseases [4,7]. In this context, naturally occurring compounds have emerged as a valuable source of PPARγ ligands [4,8]. In the context of nutraceutical research, numerous plants rich in polyphenols, flavonoids, terpenoids, and alkaloids are being studied for their potential to activate PPARγ, offering natural alternatives to synthetic medications. Additionally, bioactive fatty acids like omega-3 and omega-6, along with functional foods such as probiotics, fruits, herbs, and spices, are being explored as safer and potentially more effective options for PPARγ activation compared to synthetic drugs [9,10].

In addition to PPARs, transient receptor potential (TRP) channels play crucial roles in regulating a wide range of physiological processes, with significant implications for health and metabolism [11]. Notably, TRPA1, TRPM8, and TRPV1 serve as chemical sensors that contribute to essential cellular functions, such as ion transport and signal transduction, both of which are vital for maintaining normal cellular activities [12]. Dysfunction in these channels is linked to various metabolic diseases, given their widespread presence in metabolic tissues and involvement in metabolic pathways. TRP channels influence insulin secretion and sensitivity, thereby impacting glucose homeostasis and playing a key role in diabetes management [13]. Additionally, they are implicated in cardiovascular diseases, where they help to regulate lipid metabolism and energy balance [14]. Despite the diverse roles of PPARs and TRP channels in health and disease, challenges remain in targeting both classes of receptors and channels effectively for therapeutic purposes.

Caigua (*Cyclanthera pedata* (L.) Schrad.) is a plant that originates in the Andean region and is now cultivated in several other countries, including Italy, where it is primarily grown in the alpine valleys of the Lombardy region by small-scale farmers and hobbyist horticulturists [15]. In the alpine Camonica Valley, a local cultivar (landrace) of caigua has been documented since 1960 [16].

Caigua is valued for its therapeutic qualities in Central and South America, where it is a staple in ethnobotanical medicine practices. Widely recommended as an antidiabetic remedy [17,18], it is also endowed with antioxidant and anti-inflammatory properties [19] and is used to manage hypertension and other circulatory ailments [18]. Additionally, the fruit of this species is believed to be effective in reducing serum cholesterol levels, with a clinical study supporting this [20].

Given the traditional herbal use of caigua, the Camonica Valley landrace has attracted interest as a potential alternative source for use in herbal products, dietary supplements, and other health-oriented applications [15]. However, despite its wide use, the phytochemical properties of the plant remain largely unknown. In a recent study, we identified high levels of flavone glycosides in both fruits and leaves of this landrace, characterizing its distinctive phytochemical profile [21]. Nevertheless, comprehensive information about the phytochemical properties of its secondary metabolites is currently lacking. In this study, we document the isolation procedure of the two primary flavone glycosides from the leaves of the Camonica Valley cultivar of caigua and explore the potential biological activity of these compounds toward PPARα, β, and γ receptors, as well as TRPA1, TRPV1 and TRPM8 channels.

## 2. Results

### 2.1. Isolation of Chrysin-6-C-Fucopyranoside (1) and Isovitexin (2) from Caigua Leaves

**Figure 1:**
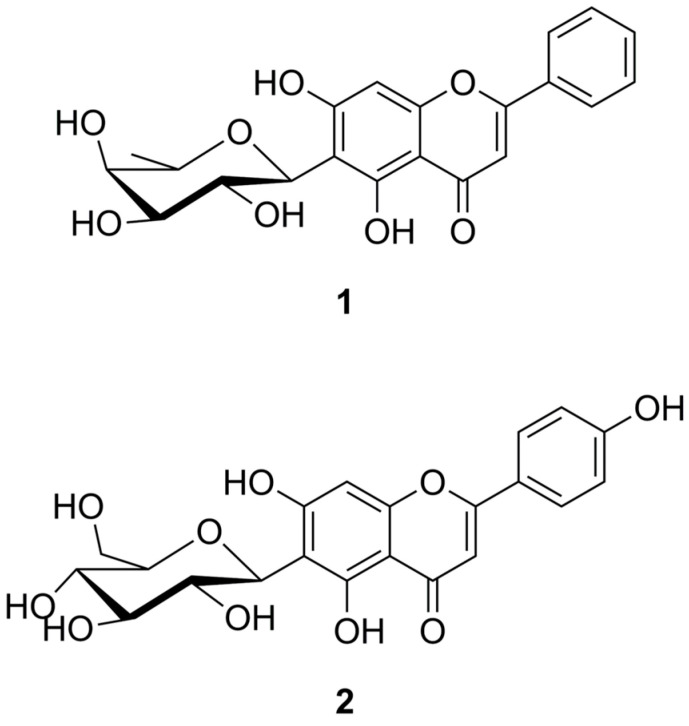
Structures of compounds chrysin-6-*C*-fucopyranoside (**1)** and isovitexin (**2**).

Two main flavone glycosides were isolated from the leaves of the Camonica Valley cultivar of caigua and identified as chrysin-6-*C*-fucopyranoside (**1**) and apigenin-6-*C*-glucopyranoside (isovitexin) (**2**). The structures of compounds **1** and **2** are shown in Figure 1.

Compounds **1** and **2** were identified by NMR and HRMS and confirmed by comparison with published data [21,22]. Spectral data are provided in Table 1.

### 2.2. Chrysin-6-C-Fucopyranoside (1) Activate PPARγ, but Not PPARα, PPARβ/δ, and TRP Ion Channel Receptors

To test the potential activity of compounds **1** and **2** toward PPAR receptors, we performed luciferase assays by transiently transfecting PPARα, PPARβ/δ, and PPARγ constructs inserted into a plasmid carrying luciferase (LUC) gene (see Section 4 for details).

Toward this goal, subconfluent COS cells (60–70%), a common fibroblast-like clonal cell line, were transiently transfected with the following plasmids:

1. PPARα, PPARγ, or PPARβ/δ (ligand binding domain –LBD)/GAL4 (dimerization binding domain –DBD);
2. UAS enhancer MH100;
3. Renilla and treated the next day with compound **1** or **2** at a concentration ranging from 0.1 to 30 µM.

**Figure 2:**
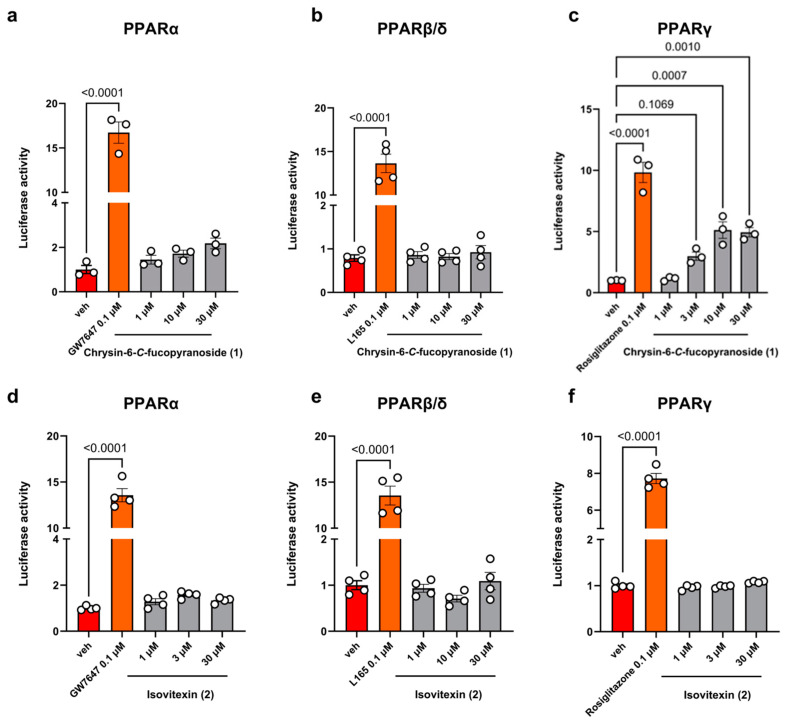
Luciferase activity of compounds **1** and **2** with PPARs. Bar Graph with individual points showing the effect of compounds **1** and **2** (from 1 to 30 µM) in COS cells transiently transfected with PPARα, PPARβ/δ, and PPARγ. Data are shown as normalised to Renilla (reported plasmid) and relative to the vehicle (DMSO) group (=1). Each bar is the mean ± standard error of the mean of three independent determinations. The selective agonists GW7647, L-165, and rosiglitazone were used as internal controls. (**a**) Luciferase activity of compound **1** on PPARα; (**b**) Luciferase activity of compound **1** on PPARβ/δ; (**c**) Luciferase activity of compound **1** on PPARγ; (**d**) Luciferase activity of compound **2** on PPARα; (**e**) Luciferase activity of compound **2** on PPARβ/δ; (**f**) Luciferase activity of compound **2** on PPARγ.

The selective agonists for PPARα (GW7647, 0.1 µM), PPARγ (rosiglitazone, 0.1 µM), and PPARβ/δ (L165, 0.1 µM) were used as internal controls of the transfection procedure. The activity data of compounds **1** and **2** toward PPARs are reported in Figure 2.

We found that compound **1** exhibited agonist activity toward PPARγ (Figure 2c), while showing no activity toward PPARα and PPARβ/δ receptors (Figure 2a,b). Conversely, compound **2** was inactive across all three receptor subtypes (Figure 2d–f).

**Figure A1:**
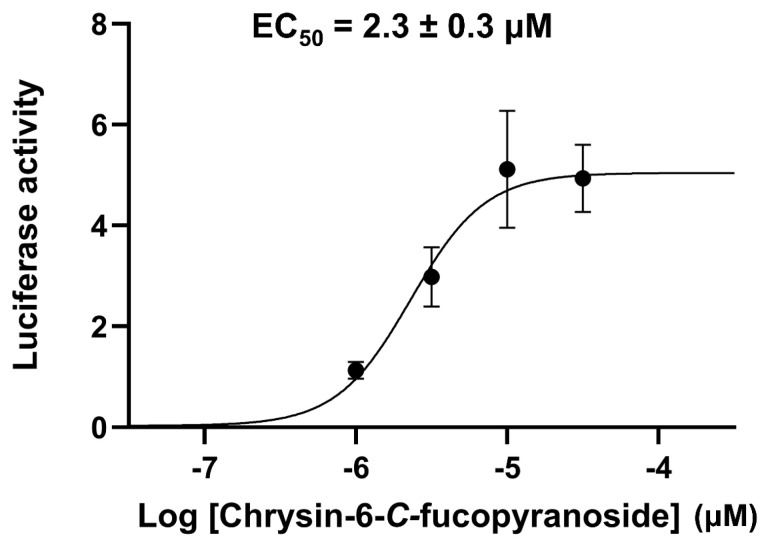
Concentration–response curve in the cell luciferase assay of compound **1** with PPARγ. Individual points showing the effect of compound **1** (from 1 to 30 µM) in COS cells transiently transfected with PPARγ. Data are shown as normalized to Renilla (reported plasmid) and relative to the vehicle (DMSO) group (=1). Each point is the mean ± SEM of three independent determinations. Efficacy at 51% was calculated on the effect of rosiglitazone.

The estimated EC_50_ value of compound **1** on PPARγ was about 2.3 µM. The EC_50_ curve and data are provided in Figure A1 and Table A1 of Appendix A.

Table 2 shows the potency value of compound **1** compared with that of other flavonoids known to activate PPAR, as reported by Salam et al. [23] and Quang et al. [24].

Therefore, comparing the EC_50_ values, compound **1** emerged as highly active, displaying an agonist potency similar in magnitude to pseudobaptigenin and formononetin (Table 2, entries 2 and 11, respectively).

**Figure 3:**
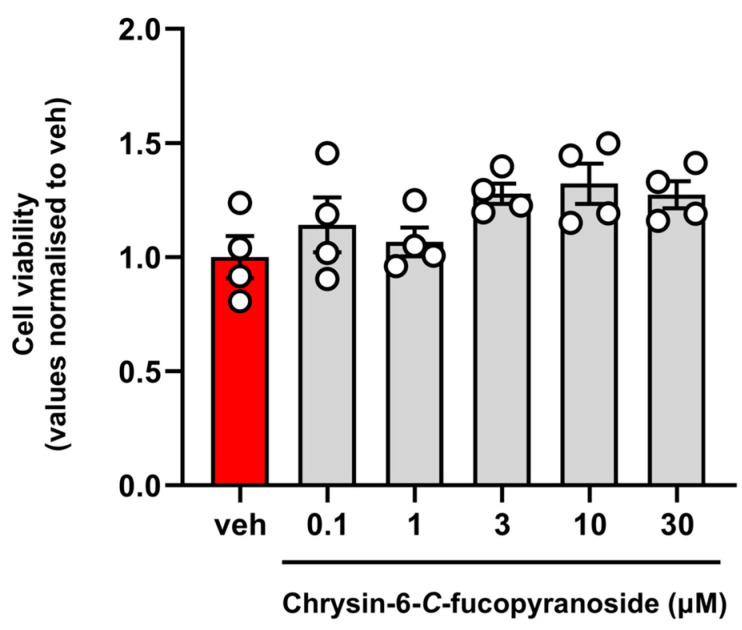
Effect of compound **1** on cell viability. The day after plating, COS-7 cells were treated with vehicle (DMSO, in red) or increasing concentrations of compound **1** (1–10 and 30 µM) for 24 h. Cell viability was assessed using the MTT assay, as described in the Section 4. Values are shown as relative to the vehicle group (set to 1) and expressed as mean ± standard error of the mean of four independent experiments.

The potential cytotoxic effects of compound **1** were next examined in COS-7 cells using the MTT assay. In these experiments, cells were incubated with increasing concentrations of compound 1 (1, 10, and 30 µM) for 24 hr. Compound **1** was found to be not toxic even at the highest concentration used (30 µM). The cytotoxicity data of compound **1** are reported in Figure 3.

Additionally, we tested the potential effects of compounds **1** and **2** on TRPA1, TRPV1 and TRPM8 ion channel receptors by performing the fluorometric calcium assay. The results obtained showed that both compounds were fully ineffective on these receptors up to 50 µM.

## 3. Discussion

Despite extensive research on PPARγ agonists for various diseases, their only approved clinical use remains as thiazolidinediones for type 2 diabetes. These agents, although effective in reducing insulin resistance, have faced market withdrawals or restrictions owing to severe side effects, including hepatotoxicity, cardiovascular risks, and the potential risk of developing bladder cancer [4]. Consequently, the search for novel selective agonists targeting PPARγ with a better therapeutic profile remains a highly active and significant area of research.

In the screening of new PPARγ agonists, the search for bioactive natural compounds is very active. Numerous papers and reviews report such activity and indicate plants and herbs as promising sources of novel antidiabetic compounds. Among these families, flavonoids appear to be one of the most interesting groups.

Salam et al. demonstrated effective PPARγ binding and activation by flavonoids through docking experiments and ELISA-based transcription factor assays. These flavonoids were benchmarked against rosiglitazone, with pseudobaptigenin and hesperidin emerging as the most potent [23]. In comparison to these natural PPARγ agonists, compound **1** exhibited remarkable activity, surpassing the potency of hesperidin and being comparable to pseudobaptigenin. Additionally, compound **1** was four times more potent than its corresponding aglycone, chrysin. Notably, chrysin has been shown to effectively induce PPARγ activation, providing protective effects in various disease models [25]. Furthermore, chrysin has proven to be a useful scaffold for designing novel PPARγ ligands [26]. The finding that compound **1** is significantly more potent presents new opportunities for the development of enhanced PPARγ ligands.

Similarly, Matin et al. and Liang et al. observed comparable results using cell-based luciferase reporter assays [27,28]. Quang et al. used a luciferase assay to measure the activity of several flavonoids toward PPARs from the roots of the medicinal plant *Sophora flavescens*. Several compounds significantly upregulated PPARγ activity in a dose-dependent manner, with EC_50_ values as low as 1.6 µM for the isoflavone formononetin [24]. These compounds are also active toward PPARα and PPARβ/δ and are therefore non-selective agonists, whereas compound **1** is a selective PPARγ agonist.

The relationship between PPARγ activity and flavonoid structure has also been explored by other investigators. Among the tested flavonoids, isoflavones exhibited high agonist activity but lacked selectivity, as in the case of formononetin. Conversely, flavone derivatives, including chrysin, though less potent, demonstrated greater selectivity toward PPARγ [27]. The role of peripheral substituents, particularly sugar residues, was explored by the same authors in an elegant study on the interaction with putative receptors and the design of pharmacophores based on a large number of flavonoids and *O*-glycosylated derivatives. In hesperidin, the highly polar sugar residue is directed toward the polar region of the receptor, making several hydrogen bonds in a docking pose similar to that of rosiglitazone. Zhou et al. identified the flavone 7-*O*-glucuronide oroxyloside as a dual agonist capable of promoting the transcriptional activity of both PPARα and PPARγ in a luciferase reporter assay [29]. Docking experiments from the same study revealed that the glucuronic acid moiety of oroxyloside forms hydrogen bonds with the amino acid residues of PPAR involved in binding with polar regions of known agonists [30].

In analogy with these two cases, the fucose moiety of compound **1** might engage in polar interactions with the receptor, suggesting a comparable binding mechanism. On the other hand, other flavone 7-*O*-glycosides, such as linarin, diosmin, neodiosmin, rhoifolin, apiin, and poncirin, were reported to be inactive toward PPARγ [27], indicating that the presence of a sugar residue is not per se a sufficient structural requisite for activity. Supporting this, the lack of activity toward PPARγ by compound **2** observed in this work compared to its aglycone apigenin further corroborates our finding, confirming that the substitution pattern of the sugar residue may be critical for receptor binding.

However, compound **1**, which is the flavone *C*-fucopyranoside, showed significant and selective PPARγ activation in our study. To the best of our knowledge, no other data on the activity of flavone-*C*-glycosides or fucosyl derivatives are currently available. Further molecular modeling and in vivo experiments are indeed essential in the future to clarify the nature of these binding and pharmacological properties in animal models of disease.

## 4. Materials and Methods

### 4.1. Extraction and Isolation of Flavone Glycosides

#### 4.1.1. General Experimental Procedures

All the solvents and reagents used were of analytical grade purity, while water was of HPLC grade purity. All of the solvents and chemicals were purchased from Merck (Milan, Italy) and used without further purification. Column chromatography was carried out on flash silica gel (Supelco 230–400 mesh), while reversed-phase column chromatography was carried out on C_18_ silica gel for column chromatography (Merck 230–400 mesh). Thin-layer chromatography (TLC) analysis was carried out on silica gel plates (Merck 60 F254) with visualization under a UV lamp (254 nm) and/or development with 1% (*m*/*v*) ethanolic solution of aluminium chloride and visualization under UV lamp (365 nm).

NMR spectra were recorded on a Bruker Avance 600 MHz spectrometer (Germany) available at the Unitech Cospect platform at the University of Milan. The NMR spectrometer was equipped with a 5 mm triple-resonance probe with *z*-axis gradients and a variable temperature control unit. The analyses were performed at 25 °C. The NMR samples were dissolved in deuterated methanol. Spectra were referenced to that of tetramethylsilane used as an external standard and taken as 0.0 ppm.

High-resolution mass spectra (HRMS) were obtained using a Synapt G2-Si QTOF mass spectrometer (Waters, USA) with a ZsprayTM ESI probe for electrospray ionization (Waters) available at the Unitech Cospect platform at the University of Milan.

#### 4.1.2. Plant Material

The leaves of the local cultivar of caigua growing in the Camonica Valley were used for this study. Plant material was provided by a local farm that cultivates this plant for commercial purposes in the municipality of Pian Camuno (latitude: 45°50′57.8″ N, longitude: 10°08′34.9″ E, elevation: 244 m a.s.l.).

The plant was confirmed to belong to the species *Cyclanthera pedata* (L.) Schrad. using DNA barcoding techniques by the center FEM2-Ambiente of the University of Milano-Bicocca [31].

Fully developed leaves were collected in September 2021. The collected plant material was prepared and dried in ventilated ovens (MPM Instruments M120-VF) at 70 °C for 36 h, as previously described [15]. The dried plant material was stored in sealed glass containers and stored in the dark at room temperature. Before extraction, the plant material was pulverized using an MM400 vibrational mill operating at 30 Hz for 60 s.

#### 4.1.3. Extraction and Isolation

The main flavone glycosides **1** and **2** were isolated from the dried leaves following the procedure reported by Zuccolo et al. [21]. Briefly, 50 g of powdered leaves were refluxed in four 20 min cycles with 200 mL of hexane for each cycle, followed by four 20 min cycles with 200 mL of chloroform for each cycle. Subsequently, the plant material was extracted three times with 500 mL of methanol at room temperature, with each extraction lasting eight hours. The combined methanol extracts were evaporated under reduced pressure at 50 °C.

The resulting crude extract was suspended in 50 mL of water and partitioned three times with 50 mL of n-butanol per cycle. The n-butanol-soluble fraction was fractionated on 30 g of Sephadex^®^ LH-20 (Merck, Germany) (30 g), using methanol as the eluent. Fractions (10 mL) were collected and combined according to their TLC profiles (eluent: chloroform/methanol (90:10) containing 1% *v/v* formic acid).

Compounds **1** and **2** were then separated from the flavone glycoside-containing fractions by flash chromatography on silica, employing mixtures of chloroform and methanol/water (11:1) containing 1% (*v*/*v*) formic acid as the eluent. Finally, the single products were purified by reversed-phase flash chromatography on C_18_ silica using methanol/water mixtures containing 0.5% (*v*/*v*) formic as the eluent.

Compound **1** (C_21_H_20_O_10_) was isolated as a yellow amorphous solid from the leaves (3.8 mg; purity 95%, HPLC) and identified as chrysin-6-*C*-fucopyranoside. Compound **2** (C_21_H_20_O_10_) was isolated as a yellow amorphous solid (2.8 mg; purity 95%, HPLC) and identified as apigenin-6-*C*-glucopyranoside (isovitexin).

Spectral data for the isolated compounds are reported in Table 1, within Section 2.1.

### 4.2. PPARs Assays. Cell Culture, Transfection, and Luciferase Assay

COS-7, a cell line exhibiting fibroblast morphology derived from the CV-1 cell line, was purchased from the American Type Culture Collection ATCC (cat. CRL-1651) and grown in DMEM supplemented with 10% fetal bovine serum and 1% penicillin/streptomycin under standard conditions. For transfection, cells were plated in 24-well dishes. The day after, at about 60–70% confluence, cells were transfected using Lipofectamine 2000 Reagent Cat# 11668019 (Life Technologies, Italy) according to the manufacturer’s instructions, with a mixture of three plasmids:

1. CMX-Gal4-hPPARα;
2. TK-MH100 × 4-Luc containing the UAS enhancer;
3. Renilla Luciferase (pRL, Promega, Cat. E2231).

Cells transfected with an equal amount of pcDNA3.1 Cat# V79020 (Life Technologies, Italy) were used as the control condition. On day 3, cells were treated for 24 h with test compounds. Rosiglitazone 0.01 μM Cat# 5325 (Tocris, UK) and GW7647 Cat# 1677 (Tocris, UK) were used as positive internal controls for PPARγ and PPARα. At the end of the treatment, the luciferase reporter activity was detected using the Dual-Glo^®^ Luciferase Assay kit Cat# E2920 (Promega, USA) using a GloMax^®^ Discover microplate reader device (Promega, USA).

### 4.3. Cell Viability Assay

COS-7 cells were plated in 24-well plates at 2 × 10^5^ cells per well. The following day, cells were treated with the test compounds for 24 h. Cells that received dimethyl sulfoxide (DMSO) (less than 0.03%), which served as the solvent for all compounds, constituted the control group (vehicle). The cells’ capability to convert 3-(4,5-dimethylthiazol-2-yl)-2,5-diphenyltetrazolium bromide (MTT) into formazan salts was considered an indicator of cell vitality, reliant on the proper functioning of mitochondria. Following a 3 h incubation period, the resulting formazan salts were solubilized in a solution containing three parts 2-methyl-2-propanol. The absorption of this solution was then measured at a wavelength of 620 nanometers using a GloMax^®^ Discover microplate reader.

### 4.4. TRP Channels Assay

Compounds **1** and **2** were tested in human embryonic kidney cells (HEK-293; ATCC CRL-1573) stably transfected with rat TRPA1, rat TRPM8, human TRPV1, or untransfected cells. Cells were grown in Minimum Essential Media (MEM Cat. n. 11095080; ThermoFisher, Milan, Italy) supplemented with non-essential amino acids (Cat. n. 11140050; ThermoFisher, Milan, Italy), 10% fetal bovine serum (FBS; Cat. n. 10270106; ThermoFisher, Milan, Italy), and 2 mM L-glutamine (Cat. n. A2916801; ThermoFisher, Milan, Italy) and maintained under 5% CO_2_ at 37 °C. Briefly, on the day of the experiment, untransfected and transfected cells were loaded with methyl ester Fluo4-AM (Cat. n. F14201; 4 µM in DMSO containing 0.02% Pluronic F-127, ThermoFisher, Milan, Italy) in MEM without FBS for 1 h in the dark at room temperature. After incubation, the cells were washed twice in Tyrode’s buffer (145 mM NaCl, 2.5 mM KCl, 1.5 mM CaCl_2_, 1.2 mM MgCl_2_, 10 mM D-glucose, and 10 mM HEPES, pH 7.4), re-suspended in Tyrode’s buffer, and transferred into the quartz cuvette of the spectrofluorimeter (Perkin-Elmer LS50B; PerkinElmer Life and Analytical Sciences, Waltham, MA, USA) under continuous stirring. Changes in the intracellular calcium concentration [Ca^2+^]_i_ were determined in the presence of increasing concentrations of compounds **1** and **2** by measuring cell fluorescence at 25 °C (λEX = 488 nm, λEM = 516 nm). Curve fitting (sigmoidal concentration-response variable slope) and parameter estimation were performed with GraphPad Prism^®^ 10 (GraphPad Software Inc., San Diego, CA, USA). The efficacy is reported as normalized to the response of 4 μM ionomycin (Cayman, USA). The signal intensity obtained in non-transfected HEK-293 cells was used as the baseline and subtracted as the background. The effects on TRPA1 are reported as a percentage of the value of 100 µM allyl isothiocyanate (AITC). Measurement of [Ca^2+^]_i_ in TRPM8 cells was performed at 22 °C using the Fluorescence Peltier System (PTP-1, Perkin-Elmer, USA). The TRPA1, TRPM8, or TRPV1 channel desensitization (reported as antagonism) was evaluated by measuring the effect of selective agonists (AITC 100 µM for TRPA1, capsaicin 0.1 µM for TRPV1, and icilin 0.25 µM for TRPM8) in the presence of compound **1** or **2** pre-incubated in the cells 5 min before stimulation.

### 4.5. Statistical Analysis

Data were analyzed using one-way ANOVA (once the assumptions of normality of group data and homogeneity of variances were verified using the Shapiro–Wilk test and Levene’s test, respectively) with the Tukey test applied post hoc. The data were expressed as mean ± standard error (SEM) and differences were considered statistically significant when *p* < 0.01. Statistical analysis was performed using R software version 4.3.1 [32].

## 5. Conclusions

Chrysin-6-*C*-fucopyranoside, isolated from a local cultivar of *Cyclanthera pedata*, has been identified as a novel and selective PPARγ agonist, indicating its potential in managing metabolic disorders like diabetes and hyperlipidemia. While PPARγ agonists are known to enhance insulin sensitivity and aid in managing type 2 diabetes, their clinical use is often restricted due to significant side effects, including an increased risk of heart failure, weight gain, bone fragility, edema, and cancer. This study is the first to demonstrate that caigua’s secondary metabolites might serve as a novel class of PPARγ modulators, expanding the traditional medicinal applications of this plant.

However, despite the novelty of this work, further research is needed to fully elucidate the bioavailability, pharmacokinetic properties, and in vivo efficacy of these compounds. The results are promising for exploiting the potential of this plant for the pharmaceutical and nutraceutical market. Caigua is a food plant and it is easily cultivated in our climate. Together with their fruit, the leaves have proven to be rich in bioactive flavonoids and constitute an important, cheap, and easily accessible biomass, available for the development of innovative healthy food products and thereby valorizing the local economy of this underexplored food plant.

## Figures and Tables

**Table 1:** ^1^H NMR (600 MHz, CD_3_OD), ^13^C NMR (150 MHz, CD_3_OD), and HRMS data for compounds **1** and **2**.

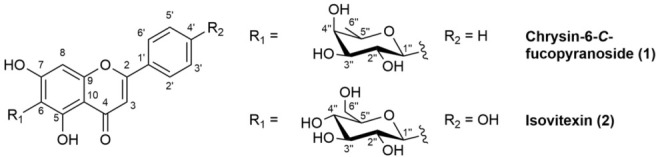				
	Compound 1		Compound 2	
	δ_H_	δ_c_	δ_H_	δ_c_
2	--	166.5	--	163.3
3	6.58 (s)	106.3	6.63 (s)	102.6
4	--	185.0	--	181.7
5	--	163.0	--	165.3
6	--	110.3	--	108.9
7	--	166.0	--	161.3
8	6.78 (s)	96.2	6.52 (s)	93.8
9	--	161.7	--	161.1
10	--	107.0	--	102.9
1′	--	131.0	--	121.0
2′	8.03 (d, *J* = 8.0 Hz) ^1^	128.3 ^1^	6.96 (d, *J* = 8.4 Hz) ^3^	128.3 ^3^
3′	7.61 (m, *J* = 8.0 Hz) ^2^	133.3 ^2^	7.88 (d, *J* = 8.5 Hz) ^4^	116.0 ^4^
4′	7.60 (m)	130.2	--	160.6
5′	7.61 (m, *J* = 8.0 Hz) ^2^	133.3 ^2^	7.88 (m, *J* = 8.5 Hz) ^4^	116.0 ^4^
6′	8.03 (d, *J* = 8.0 Hz) ^1^	128.3 ^1^	6.96 (d, *J* = 8.4 Hz) ^3^	128.3 ^3^
1″	4.96 (bs)	76.2	4.93 (d, *J* = 9.8 Hz)	73.1
2″	4.73 (m)	72.6	4.17 (m)	70.5
3″	3.65 (dd, *J* = 8.9; 3.6 Hz)	83.5	3.51 (m)	78.9
4″	3.79 (m)	73.5	3.48 (m)	70.2
5″	3.85 (m)	81.0	3.42 (m)	81.4
6″	1.28 (d, *J* = 6.5 Hz)	17.6	3.87 (m) 3.74 (m)	61.4
HRESIMS	*m*/*z* 401.1111 [M + H]^+^ (calcd for C_21_H_20_O_8_, 400.1158)		*m*/*z* 432.01017 [M]^+^ (calcd for C_21_H_20_O_10_, 432.1056)	

^1–4^ Overlapping signals.

**Table 2:** EC_50_ values for transcriptional factor activity of compound **1** in comparison with the EC_50_ of flavonoids reported by Salam et al. [23], and Quang et al. [24]. All results are expressed as mean ± standard error of the mean.

Entry	Compound	EC_50_ (μM)	
1	Chrysin-6-*C*-fucopyranoside (**1**)	2.3 ± 0.3	Present work
2	Pseudobaptigenin	2.9 ± 0.8	[23]
3	Esperidin	6.6 ± 1.2	
4	Apigenin	7.9 ± 1.4	
5	Chrysin	9.8 ± 3.3	
6	Biochanin A	9.6 ± 1.1	
7	Genistein	16.7 ± 0.8	
8	(2R)-3α,7,4′-trihydroxy-5-methoxy-8-(γ,γ-dimethylallyl)-flavanone	10.5 ± 2.0	[24]
9	Norkurarinone	6.6 ± 1.1	
10	Kuraridin	15.7 ± 1.0	
11	Formononetin	1.6 ± 0.5	

## Data Availability

The data supporting the results of this study can be obtained from the corresponding authors upon reasonable request.

## Abbreviations

The following abbreviations are used in this manuscript:

AITC	Allyl isothiocyanate
ANOVA	Analysis of variance
DBD	Dimerization binding domain
DMSO	Dimethyl sulfoxide
DMEM	Dulbecco’s modified Eagle medium
EC_50_	Half-maximal effective concentration
ELISA	Enzyme-linked immunosorbent assay
FBS	Fetal bovine serum
Fluo4-AM	Methyl ester of fluo-4 acetoxymethyl ester
HEK-293	Human embryonic kidney 293 cells
LBD	Ligand binding domain
LUC	Luciferase
MEM	Minimum essential media
MTT	3-(4,5-Dimethylthiazol-2-yl)-2,5-diphenyltetrazolium bromide
PPAR	Peroxisome proliferator-activated receptor
PPARα	Peroxisome proliferator-activated receptor alpha
PPARβ/δ	Peroxisome proliferator-activated receptor beta/delta
PPARγ	Peroxisome proliferator-activated receptor gamma
pRL	Renilla luciferase plasmid
SE	Standard error
SEM	Standard error of the mean
TK-MH100 × 4-LUC	Thymidine kinase promoter with four multimerized MH100 enhancer elements
TRP	Transient receptor potential cation channel
TRPA1	Transient receptor potential subfamily A member 1
TRPM8	Transient receptor potential subfamily M member 8
TRPV1	Transient receptor potential subfamily V member 1
UAS	Upstream activating sequence
λEX	Excitation wavelength
λEM	Emission wavelength

## Appendix A

EC_50_ curve and data for compound **1** are provided in Figure A1 and Table A1.

**Table A1:** Concentration–response data in the cell luciferase assay for compound **1** with PPARγ. Effect of compound **1** (from 1 to 30 µM) in COS cells transiently transfected with PPARγ. Data are shown as normalized to Renilla (reported plasmid) and relative to the vehicle (DMSO) group (=1). Rosiglitazone at 0.1 μM was used as the internal standard.

Treatment	Vehicle	Compound 1				
		0.1 μM	1 μM	3 μM	10 μM	30 μM
Replicate 1	1.03	1.09	1.27	3.61	5.19	5.66
Replicate 2	0.98	0.82	0.94	2.45	3.92	4.79
Replicate 3	0.98	1.04	1.19	2.88	6.25	4.36
Mean ± SD	1.00 ± 0.03	0.98 ± 0.14	1.13 ± 0.17	2.98 ± 0.59	5.12 ± 1.16	4.94 ± 0.67
